# An Intersectional Viral-Genetic Method for Fluorescent Tracing of Axon Collaterals Reveals Details of Noradrenergic Locus Coeruleus Structure

**DOI:** 10.1523/ENEURO.0010-20.2020

**Published:** 2020-05-15

**Authors:** Nicholas W. Plummer, Daniel J. Chandler, Jeanne M. Powell, Erica L. Scappini, Barry D. Waterhouse, Patricia Jensen

**Affiliations:** 1 Neurobiology Laboratory, National Institute of Environmental Health Sciences, National Institutes of Health, Department of Health and Human Services, Research Triangle Park, NC 27709; 2 Department of Cell Biology and Neuroscience, Rowan University School of Osteopathic Medicine, Stratford, NJ 08084; 3 Signal Transduction Laboratory, National Institute of Environmental Health Sciences, National Institutes of Health, Department of Health and Human Services, Research Triangle Park, NC 27709

**Keywords:** axon tracing, CAV2, locus coeruleus, noradrenergic, norepinephrine, recombinase

## Abstract

Understanding the function of broadly projecting neurons depends on comprehensive knowledge of the distribution and targets of their axon collaterals. While retrograde tracers and, more recently, retrograde viral vectors have been used to identify efferent projections, they have limited ability to reveal the full pattern of axon collaterals from complex, heterogeneous neuronal populations. Here we describe TrAC (tracing axon collaterals), an intersectional recombinase-based viral-genetic strategy that allows simultaneous visualization of axons from a genetically defined neuronal population and a projection-based subpopulation. To test this new method, we have applied TrAC to analysis of locus coeruleus norepinephrine (LC-NE)-containing neurons projecting to medial prefrontal cortex (mPFC) and primary motor cortex (M1) in laboratory mice. TrAC allowed us to label each projection-based LC-NE subpopulation, together with all remaining LC-NE neurons, in isolation from other noradrenergic populations. This analysis revealed mPFC-projecting and M1-projecting LC-NE subpopulations differ from each other and from the LC as a whole in their patterns of axon collateralization. Thus, TrAC complements and extends existing axon tracing methods by permitting analyses that have not previously been possible with complex genetically defined neuronal populations.

## Significance Statement

We have developed a new method for mapping axon collaterals of genetically defined neuronal subtypes. TrAC (tracing axon collaterals) uses an intersectional genetic strategy to define a cell population of interest, and a retrograde viral construct to label a subpopulation based on its axonal projections. This method has three major benefits: (1) only one viral injection is required for labeling, (2) axons from a projection-based subpopulation can be visualized together with a broader genetically defined population, and (3) the cell population of interest can be defined by transient developmental genetic information. Our proof-of-principle analysis of noradrenergic locus coeruleus (LC) projections to the forebrain extends previous analyses and reveals new details of this complex system.

## Introduction

Fundamental to our understanding of neural circuit dynamics and function is comprehensive knowledge of the regions of the CNS innervated by neurochemically identified cell types. While conventional anatomic tract tracing methods have long been used to identify the afferent inputs and efferent projections of neuronal populations, these traditional approaches have proven impractical for revealing the full distribution of axon collaterals from a broadly projecting pool of neurons. One such brain region is the noradrenergic nucleus locus coeruleus (LC), a brainstem nucleus which provides norepinephrine (NE) to virtually the entire CNS. Early investigations into LC anatomy using conventional tracing methods suggested that its cells possess highly ramified axons with the potential to innervate broad regions of the CNS without regard for terminal field function (Bloom et al., 1971; Foote et al., 1980, 1983; Aston-Jones et al., 1986; Loughlin et al., 1986). Because of this apparent lack of functional organization, the LC has long been considered a homogeneous entity with neurons that release NE globally, thus promoting uniform actions throughout the brain and spinal cord simultaneously.

More recent evidence suggests that individual LC neurons innervate terminal fields in a more discrete and orderly fashion than originally thought (Simpson et al., 1997; Howorth et al., 2009; Chandler et al., 2014, 2019; Uematsu et al., 2015, 2017; Bellesi et al., 2016; Li et al., 2016; Hirschberg et al., 2017; McCall et al., 2017; Plummer et al., 2017). For example, it has been shown that LC cells projecting to medial prefrontal cortex (mPFC) are anatomically, molecularly, and electrophysiologically distinct from those that project to primary motor cortex (M1; Chandler et al., 2013, 2014). Additionally, injection of retrograde tracers into functionally related terminal fields (e.g., structures along the ascending somatosensory pathway) results in a higher percentage of double-labeled LC-NE neurons than when tracers are injected into functionally unrelated terminal fields (Simpson et al., 1997).

Based on these findings, there is renewed interest in identifying the collateral projections of the LC-NE system. Several laboratories have attempted to define the organizational principles of this nucleus using a variety of novel approaches that have yielded conflicting results. While some have provided evidence for a modularly organized LC with subpopulations of neurons possessing limited axonal arborization confined to a small number of terminal fields (Chandler et al., 2013, 2014; Kebschull et al., 2016; Hirschberg et al., 2017; Uematsu et al., 2017), others have reported highly collateralized LC axons that innervate many functionally unrelated efferent targets (Schwarz et al., 2015). In order to resolve these discrepancies and gain a better understanding of the functional circuit-level organization of the LC, it is necessary to develop and apply methods that can faithfully identify the CNS regions that receive collateral projections from LC-NE neurons projecting to a defined target. In a system this complex, with other brainstem noradrenergic neuron populations projecting to many of the same target sites, a method is required that can account for all LC-NE neurons without labeling any other NE neuron populations.

Toward this goal, we developed a methodology termed TrAC (tracing axon collaterals) that combines intersectional recombinase-based genetics with retrograde viral delivery of a recombinase to label axon collaterals from genetically defined cell types. Here, we describe TrAC and its application to reveal collateral networks of LC-NE neurons projecting to mPFC or M1, demonstrating the utility of this approach for dissecting the anatomy of complex, neurochemically identified neuronal populations. We found that retrograde viral delivery of a recombinase into either terminal field region produced similar numbers and distributions of retrogradely labeled LC neurons, but the patterns of axon collateralization were not identical. The labeled neurons had dense projections to the site of injection, with widespread, albeit sparse, collateral axons present in other cortical and subcortical targets. These findings are in general agreement with many other studies, suggesting that the LC-NE efferent pathway is functionally ordered and modular in design.

## Materials and Methods

### Animals

This study was performed in accordance with the recommendations in the Guide for the Care and Use of Laboratory animals of the National Institutes of Health. The protocols were approved by the Animal Care and Use Committees (ACUC) of the National Institute of Environmental Sciences and Rowan University.

To fluorescently label LC-NE neurons with tdTomato, B6.Cg-*Gt(ROSA)26Sor^tm1.1(CAG-tdTomato,-EGFP)Pjen^* mice (*RC::RFLTG*; The Jackson Laboratory stock no. 026930; Plummer et al., 2015) were intercrossed with B6.129-*En1^tm1.1(dreo)Pjen^* (*En1^Dre^*; The Jackson Laboratory Stock no. 033953; Plummer et al., 2016) and B6;129-*Dbh^tm1(flpo)Pjen^* mice (*Dbh^Flpo^*; The Jackson Laboratory stock no. 033952; Robertson et al., 2013), generating triple heterozygotes. To subsequently label subpopulations of those LC-NE neurons with EGFP based on their axonal projection pattern, *En1^Dre^; Dbh^Flpo^; RC::RFLTG* triple heterozygotes were stereotaxically injected in mPFC or M1 with the CAV2-Cre canine adenoviral vector (Hnasko et al., 2006).

### Surgery

Male and female mice were deeply anesthetized through isoflurane inhalation (4%) and placed in a stereotaxic frame. Isoflurane concentration was decreased to 2.5% after reaching a surgical plane of anesthesia. Body temperature was monitored and controlled throughout the surgical procedure. Surgery was performed in flat skull orientation such that bregma and λ were level. The dorsoventral coordinate position of each was measured, and if they were found to be >0.1 mm apart, the position of the nose cone was adjusted until they were level. Stereotaxic coordinates for viral infusions were as follows, in mm, from bregma: mPFC: AP +1.95, ML +0.9, DV −2.5 at 15° from the brain surface; M1: AP +0.26, ML +1.2, DV −0.63 from the brain surface. All infusions were made into the left hemisphere. After drilling a small craniotomy above either region, a 31-gauge Hamilton 1-μl Neuros syringe (part #7001) mounted on a World Precision Instruments stereotaxic syringe pump (model UMP3) was gradually lowered into the brain; 0.2-μl CAV2-CMV-Cre (5.6 × 1012 pfu/ml) was injected into either region at a flow rate of 0.5 μl/min using a World Precision Instruments Micro4 MicroSyringe Pump Controller. The needle remained in place undisturbed for 10 min on completion of the injection before being gradually withdrawn. Craniotomies were then filled with sterile bone wax and the incision was closed with wound clips. After surgery was complete, mice were returned to their home cages.

### Tissue collection

Six weeks after viral injection, mice were anesthetized with sodium pentobarbital and transcardially perfused with 4% paraformaldehyde (PFA) in 0.01 m PBS. Dissected brains were postfixed by immersion in 4% PFA at 4°C. Brains to be sectioned were marked on the uninjected hemisphere with black TMD Tissue Marking Dye (General Data Company), equilibrated in 30% sucrose in PBS at 4°C, and embedded in Tissue Freezing Medium (General Data Company). Free-floating 40-μm sections were collected in PBS and then stored at −80°C in 30% sucrose/30% ethylene glycol in PBS. For the passive clarity technique (PACT; Yang et al., 2014), 3-mm-thick slices containing the LC were cut using a stainless-steel coronal brain matrix (Zivic Instruments), and the remaining forebrain was embedded for sectioning as described above. PACT tissue clearing was performed as previously described (Plummer et al., 2015, 2017).

### Immunohistochemistry

We used immunofluorescent labeling to enhance signal from fluorescent proteins in fixed tissue. For simultaneous detection of virally transduced (EGFP-labeled) and non-transduced (tdTomato-labeled) LC-NE axons, a chicken anti-GFP antibody (1:10,000; catalog #ab13970, Abcam) was used in conjunction with Alexa Fluor 488 goat anti-chicken secondary antibody (1:1000; catalog #A11039, Thermo Fisher Scientific), and a rabbit anti-dsRed antibody (1:500; catalog #632496, Clontech Laboratories) was used with Alexa Fluor 568 goat anti-rabbit secondary antibody (1:1000; catalog #A11036, Thermo Fisher Scientific). Mouse monoclonal anti-NET antibody (1:1000; clone NET-05; catalog #1447-NET, PhosphoSolutions; Matthies et al., 2009) was used with Alexa Fluor 633 goat anti-mouse secondary antibody (1:1000; catalog #A21052, Thermo Fisher Scientific). Tissue was incubated with primary antibodies for 2 d at 4°C and with secondary antibodies for 2 h at room temperature. Following antibody incubations, sections were incubated for 1 h in a 1:50 dilution of Neurotrace 435/455 Blue Fluorescent Nissl Stain (Thermo Fisher Scientific) for detection of neuronal cell bodies.

For immunofluorescent labeling after PACT tissue clearing, we used chicken anti-GFP (1:1000) and rabbit anti-dsRed (1:500) primary antibodies, followed by Alexa Fluor 488 donkey anti-chicken F(ab’)2 fragments (1:500; catalog #703-546-155, Jackson ImmunoResearch) and Alexa Fluor 568 donkey anti-rabbit F(ab’)2 fragments (1:500; catalog #Ab175694, Abcam). Incubations were performed at room temperature for 6 d, with antibody replaced by fresh solution after 3 d.

### Digital image collection

LC-NE neurons in PACT-cleared tissue or 40-μm sections were imaged on an LSM 780 or 880 inverted confocal microscope (Carl Zeiss Microscopy), using an EC Plan-Neuofluar 10×/0.30 M27 objective. For imaging axonal fibers, z-stack images through the full thickness of each 40-μm section were collected on an LSM 880 confocal microscope using a PLAN Apochromat 20×/0.8 M27 or Plan Aprochromat 40×/1.3 Oil M27 objective. Alexa Fluor 633 fluorescence was excited with a 633-nm laser (pinhole setting 1 airy unit), and fluorescent emission was collected with a 640- to 758-nm filter. Alexa Fluor 568 fluorescence was excited with a 561-nm laser and collected with a 571- to 633-nm filter. Alexa Fluor 488 was excited with a 488-nm laser and collected with a 491- to 562-nm filter, and also with a 571- to 633-nm filter for an autofluorescence-only channel to permit DEFiNE image processing (Powell et al., 2019). Blue Nissl fluorescence was excited with a 405-nm laser and collected with a 415- to 47- nm filter. For fiber quantification, each anatomic region was imaged from at least three sections. The investigator was blind to labeled fibers during selection of the regions to be imaged. Location was determined by external marking with Tissue Marking Dye (see above, Tissue collection) and by anatomy, as revealed by labeling with Nissl stain, compared with a mouse brain atlas (Paxinos and Franklin, 2013).

### Image processing and quantification of LC neurons and axons

LC neurons were quantified from a total of four mice for each injection site (from 40-μm sections, *n* = 2 mPFC injections and *n* = 1 M1 injection; and from 40-μm “virtual” sections of PACT-cleared tissue, *n* = 2 mPFC injections and *n* = 3 M1 injections). For counting LC-NE neurons in 40-μm sections, z-stack images were collected from every fourth section through the full rostrocaudal extent of the fluorescently labeled LC. Maximum-intensity projections (MIPs) were imported into FIJI software (Schindelin et al., 2012) for counting. Images collected from tissue cleared by PACT were viewed with Imaris software (Bitplane) for three-dimensional rendering of the entire region and collection of 40-μm virtual sections using the Ortho Slicer function. Virtual sections spaced 160 μm apart to match the sectioned tissue were imported into FIJI for cell counting.

For axon quantification, z-stack images collected with the 20× objective were first cropped to the center six z-slices to ensure that the same volume of tissue would be compared in all images and to exclude autofluorescent particles such as dust or secondary antibody conglomerates lying between the tissue and coverslip; 400 × 400 μm areas from larger images were selected for quantification, again using Nissl stain for identification of anatomic regions. This fixed size was chosen to maximize the volume quantified while remaining within the boundaries of regions defined by the mouse brain atlas (Paxinos and Franklin, 2013). To minimize the effects of possible variation in axon density within an anatomic brain region, axons were quantified in three sections from each region.

Before quantification, images were processed using the DEFiNE macro for FIJI (Powell et al., 2019) to remove autofluorescence and other fluorescent artifacts which would affect fiber density measurements. Images used as illustrations in figures were further processed only by adjusting brightness and contrast across the entire image. After conversion of the processed z-stack to a MIP, the DEFiNE Quantify Fibers function (Powell et al., 2019) was used to determine the relative density of GFP-labeled and tdTomato-labeled axons. The processed images were converted to binary at a threshold cutoff of 4 SDs above mean background pixel intensity, and the quantity of labeled axonal fibers was calculated as the sum of pixels remaining in the three images from each anatomic region. This calculation thus reflects both fiber density and caliber. To quantify the GFP-labeled axons as a percentage of total LC inputs to a region, the number of GFP pixels was divided by the sum of GFP and tdTomato pixels. Fractional innervation by the retrogradely labeled subpopulations across anatomic regions was calculated as the number of GFP pixels in a particular region divided by the number of GFP pixels in all regions imaged (Schwarz et al., 2015). Similarly, fractional innervation by the LC as a whole was calculated as the sum of GFP and tdTomato pixels in a region divided by the sum of GFP and tdTomato pixels in all regions.

### Statistical analysis and graphing

All data are expressed as the mean ± SEM. Unpaired *t* tests, two-way ANOVA, and graphing were performed using GraphPad Prism 7 (GraphPad Software Inc.) and Microsoft Excel (Microsoft Corporation). The number of subjects and specific statistical analyses used in each experiment are indicated in the text and figure legends.

## Results

**Figure 1.**
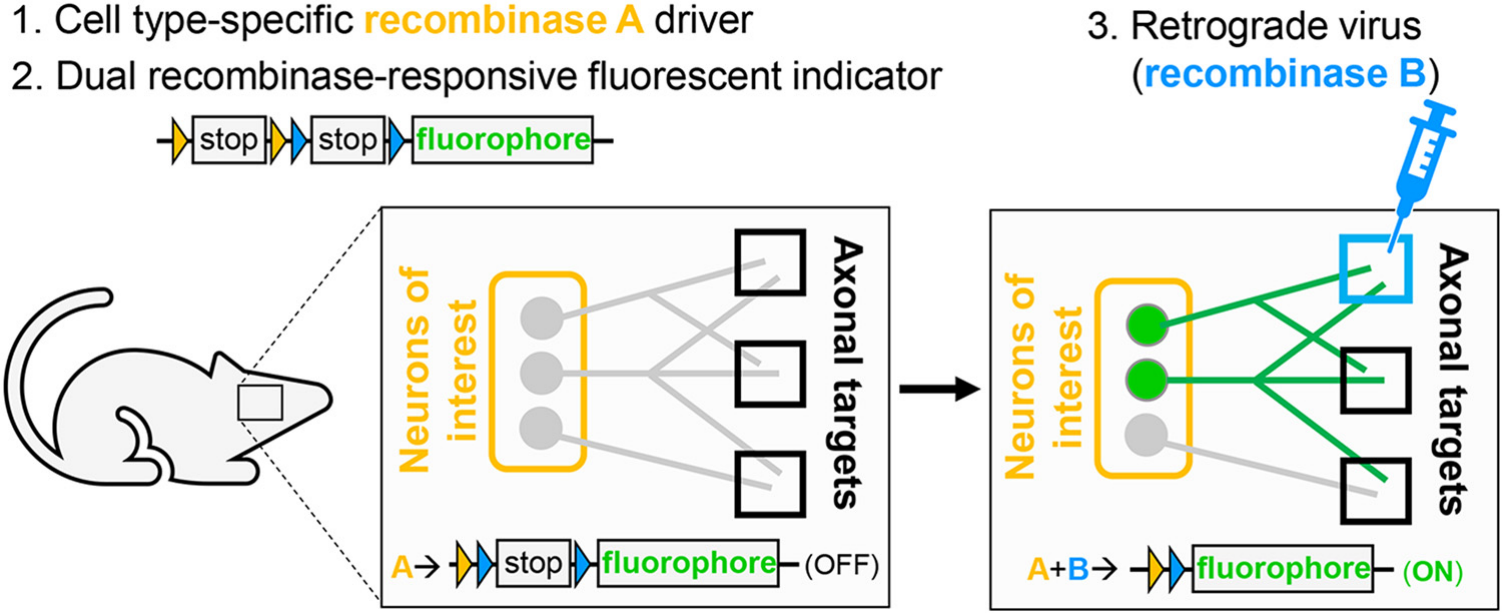
TrAC permits fluorescent labeling of genetically defined neuron populations based on axonal projections. Schematic diagrams show a simple version of TrAC using two recombinases and a dual-recombinase-responsive fluorescent indicator allele. Fluorescent labeling is restricted to neurons expressing recombinase A which project to the brain region injected with the retrograde-transported virus encoding recombinase B.

Current viral genetic strategies for mapping efferent projections obtain cellular specificity via injection of two viruses, one into the target region and a second in the cell population of interest, increasing the chance of variable targeting. To remove one of these variables and allow reproducible targeting of genetically defined populations of neurons, we developed the TrAC strategy. TrAC requires three basic elements: (1) a cell-type-specific recombinase driver allele, (2) a dual or triple recombinase-responsive fluorescent indicator allele capable of labeling axons on recombinase activation, and (3) a virus for retrograde delivery of one of the recombinases (Fig. 1). When combining these elements, the virally delivered recombinase is injected into the target region of interest of a transgenic mouse expressing the cell-type-specific recombinase(s) and dual or triple recombinase-responsive fluorescent indicator (Fig. 1). Upon recombination of the indicator allele by the retrogradely delivered recombinase, only genetically defined cells projecting to that target region as well as their axons and collaterals will be marked by the fluorescent reporter (Fig. 1).

**Figure 2.**
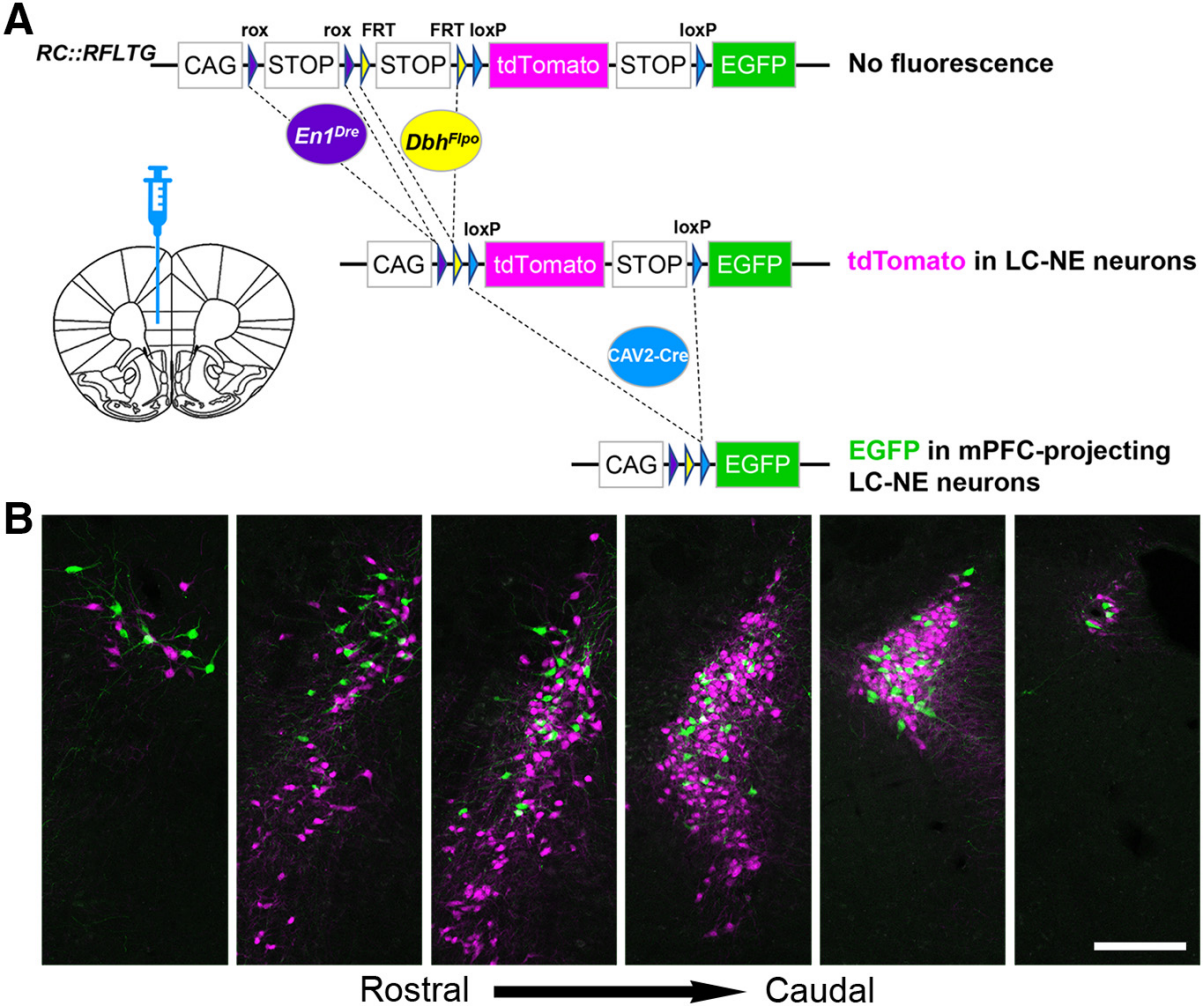
Location of mPFC-protecting noradrenergic neurons within the LC. ***A***, Schematic diagram of the *RC::RFLTG* indicator allele and coronal schematic of mouse forebrain section showing position of CAV2-Cre injection. ***B***, Representative coronal sections through the rostrocaudal extent of the ipsilateral LC from a TrAC-LC mouse (40-μm free-floating sections) showing distribution of EGFP-labeled (green) and tdTomato-labeled (magenta) neurons. Scale bar, 200 μm.

To apply TrAC to the LC-NE system, we needed a way to label LC-NE neurons in isolation from other noradrenergic neuron populations. The anatomically defined LC is located in the central gray of the pons adjacent to the lateral edge of the fourth ventricle (Grzanna and Molliver, 1980). Ventrally, the more scattered neurons of the subcoeruleus, separated into dorsal and ventral subdivisions by their position relative to the motor nucleus of the trigeminal, form a continuum between the LC and the more rostral A7 and ventral A5 noradrenergic nuclei (Aston-Jones, 2004; Paxinos and Franklin, 2013). We exploited the fact that 99.6% of the anatomically defined LC, together with smaller portions of the dorsal subcoeruleus and A7 immediately adjacent to and continuous with the LC, share a history of the transcription factor engrailed-1 (*En1*) and the noradrenergic marker dopamine-β-hydroxylase (*Dbh*; Robertson et al., 2013). In contrast to viral targeting, use of this genetic classification allows restricted and reproducible targeting of a subpopulation of noradrenergic neurons that closely matches the anatomically defined LC. To label the genetically defined LC-NE neurons in mice, we crossed *En1^Dre^* and *Dbh^Flpo^* to a triple recombinase-responsive indicator (*RC::RFLTG*; Plummer et al., 2015). In *En1^Dre^; Dbh^Flpo^; RC::RFLTG* triple transgenic (TrAC-LC) mice, tdTomato expression is restricted to genetically defined LC-NE neurons, switching to EGFP only on retrograde delivery of Cre recombinase (Fig. 2*A*).

To identify the LC-NE neurons projecting to the mPFC, we injected canine adenovirus encoding Cre recombinase (CAV2-CMV-Cre) into the mPFC of TrAC-LC and Flp-negative littermate control mice and examined fluorescence (Fig. 2*A*). We found mPFC-projecting NE neurons (EGFP+) intermingled among tdTomato+ NE neurons within the LC (Fig. 2*B*). Ipsilateral to the injection site, we counted an average of 140 ± 16 mPFC-projecting (EGFP+) and 476 ± 18 tdTomato+ NE neurons, indicating that CAV2-Cre injection in the mPFC labeled 22.54 ± 1.9% of the ipsilateral LC. Contralateral to the injection site, we observed much sparser labeling, with 20 ± 6 mPFC-projecting (EGFP+) NE neurons intermingled among 519 ± 47 tdTomato+, indicating that the CAV2-Cre labeled 3.83 ± 1.3% of the contralateral LC. Thus, 87.5% (140/160) of the retrogradely labeled neurons were ipsilateral to the injection site, and 12.5% (20/160) were contralateral to the injection site. This distribution is similar to that reported in previously published reports of LC projections to cortical targets using conventional retrograde tracer techniques (Simpson et al., 1997; Chandler and Waterhouse, 2012; Chandler et al., 2014).

To investigate the distribution of EGFP+ retrogradely labeled mPFC-projecting NE neurons within the LC, we quantified them as a percentage of all labeled neurons (EGFP+ tdTomato) in three anatomic domains: the caudal core of the anatomically defined LC, the rostral extension of the anatomically defined LC (Grzanna and Molliver 1980), and the subcoeruleus/A7. Ipsilateral to the injection site, EGFP+ mPFC-projecting neurons were distributed throughout the rostrocaudal extent of the anatomically defined LC, but they constituted a greater percentage of the rostral extension than the core (37.13 ± 4.67% rostral extension, 22.55 ± 2.90% core; *p* = 0.0378^a^, unpaired *t* test; Table 1). In contrast, EGFP+ mPFC-projecting NE neurons were generally absent from the anatomically defined subcoeruleus/A7 (two mice had one EGFP+ neuron). Although there were far fewer EGFP+ neurons in the contralateral anatomically defined LC, compared with the ipsilateral, their rostrocaudal distribution appeared to be the same. As expected, no EGFP+ NE-neurons were observed in Flp-negative littermate control mice.

**Table 1:** Statistical table

	Data structure	Type of test	95% confidence interval of difference
a	Normal	Unpaired *t* test	1.144 to 28.02
b	Normal	Unpaired *t* test	–16.90 to 17.99
c	Normal	Two-way repeated measures ANOVA (Bonferroni *post hoc* test)	0.004639 to 0.1114
d	Normal	Two-way repeated measures ANOVA (Bonferroni *post hoc* test)	0.08626 to 0.1930
e	Normal	Unpaired *t* test	0.006947 to 0.1453

See also Figure 6 legend.

**Figure 3.**
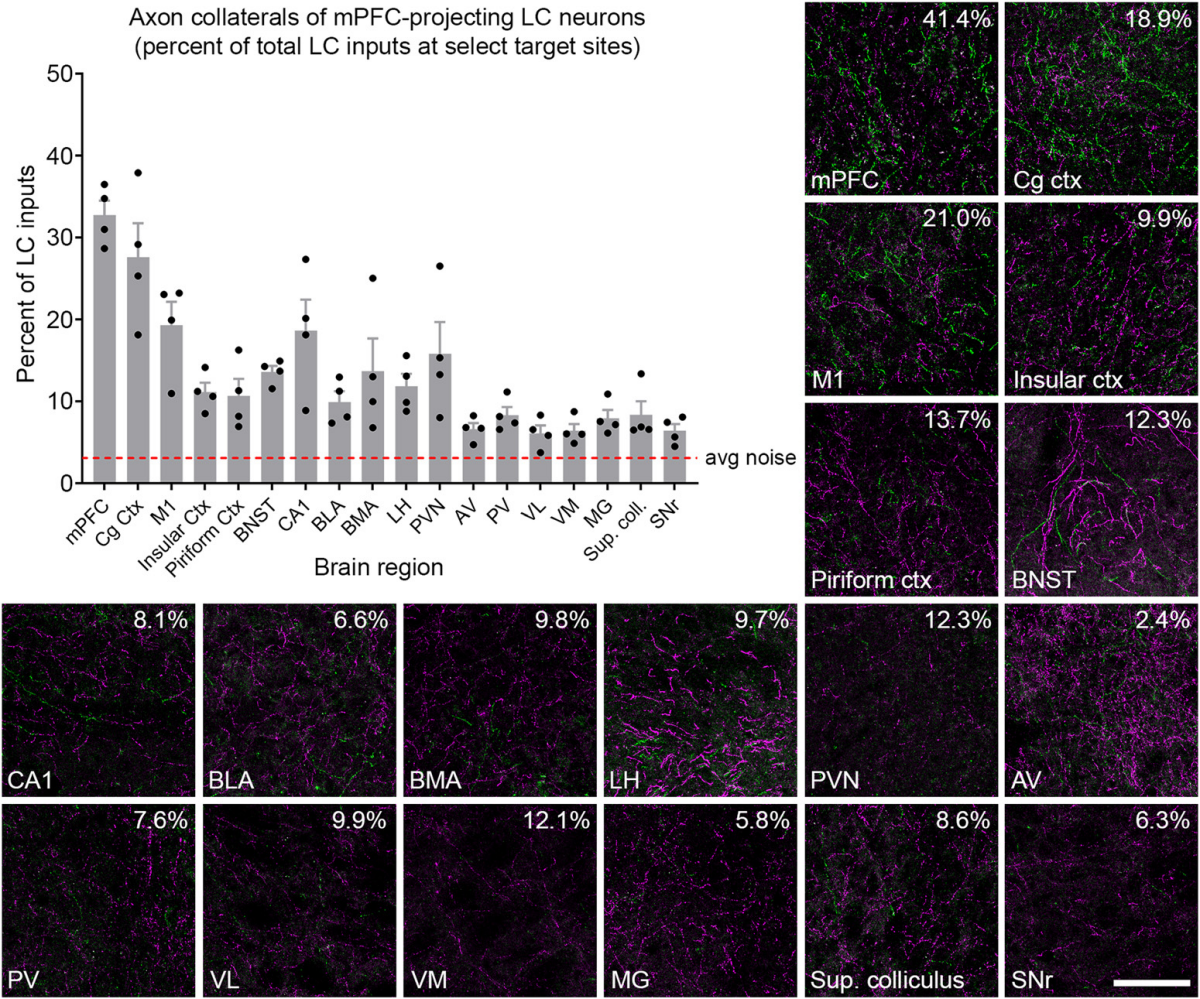
Distribution of axon collaterals from LC neurons projecting to mPFC. Representative fluorescent images show Cre+ (EGFP-labeled, green) and Cre-negative (tdTomato-labeled, magenta) axons in select forebrain and midbrain regions of a TrAC-LC mouse injected with CAV2-Cre in mPFC. Numbers in the upper right corner of each image indicate the percent of LC-NE axons in the image that are EGFP+. The bar graph (*n* = 4 mice) indicates percentage of LC-NE axons at each brain region that originate from EGFP+ mPFC-projecting LC-NE neurons (mean ± SEM). The dotted line represents the noise in the green channel (average green pixel count in areas qualitatively lacking EGFP-labeled axons divided by average total pixel count). Percentages below the dotted line are indistinguishable from noise. The scale bar for fluorescent images indicates 100 μm, and each image represents 13% of the volume quantified in the bar graph. mPFC, medial prefrontal cortex; Cg Ctx, cingulate cortex; M1, primary motor cortex; BNST, bed nucleus of the stria terminalis; CA1, area CA1 of the hippocampus; BLA, basolateral amygdala; BMA, basomedial amygdala; LH, lateral hypothalamus; PVN, paraventricular hypothalamic nucleus; AV, anteroventral thalamic nucleus; PV, paraventricular thalamic nucleus; VL, ventrolateral thalamic nucleus; VM, ventromedial thalamic nucleus; MG, medial geniculate nucleus; Sup. coll., superior colliculus; SNr, substantia nigra.

Next, we analyzed the distribution of axons and collaterals from the retrogradely labeled mPFC-projecting LC-NE neurons in select brain regions (Fig. 3). We confirmed noradrenergic identity of the fluorescently labeled axons by immunostaining with an anti-NE transporter (NET) antibody (data not shown). EGFP+ collaterals were widespread in multiple cortical regions, including the motor, cingulate, insular, and piriform cortices. In addition, we observed collaterals in multiple subcortical regions, including the bed nucleus of the stria terminalis (BNST), hippocampus, amygdala, hypothalamus, and some thalamic nuclei. Because LC-NE neurons not infected by the CAV2-Cre virus express tdTomato in TrAC-LC mice, we were able to quantify axon collaterals from EGFP+ mPFC-projecting LC-NE neurons as a percentage of all LC-NE inputs to each target region, an analysis that was not possible in prior retrograde viral studies of LC projections (Schwarz et al., 2015; Uematsu et al., 2017). The contribution from EGFP+ neurons was greatest in mPFC close to the injection site (32.7 ± 1.8%). At other target sites that we examined, the percent contribution from the EGP+ mPFC-projecting LC-NE neurons varied from 27.6 ± 0.041% in cingulate cortex (Cg ctx) to 6.1 ± 0.9% in ventrolateral thalamic nucleus (VL; Fig. 3). Outside the cortex, we observed the highest percent contribution in the hippocampus (CA1; 18.6 ± 3.8%) and paraventricular hypothalamic nucleus (PVN; 15.8 ± 3.9%). Percent contribution in thalamic nuclei was generally low, but was consistently above the average noise in the images. (Fig. 3).

**Figure 4.**
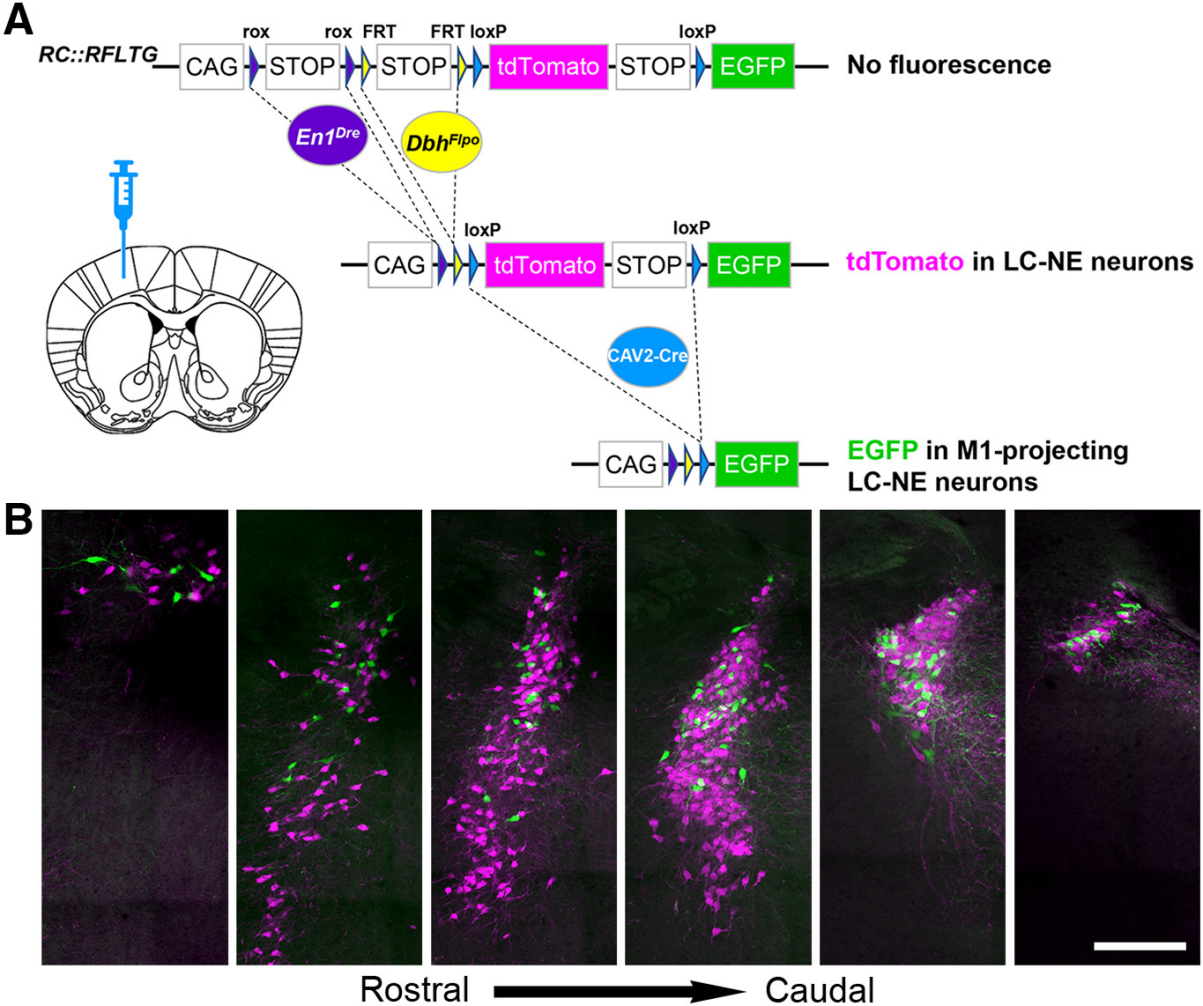
Location of motor cortex-projecting noradrenergic neurons within the LC. ***A***, Coronal schematic of mouse forebrain section showing position of CAV2-Cre injection. ***B***, Representative coronal sections through the rostrocaudal extent of the ipsilateral LC from a TrAC-LC mouse (40-μm virtual sections from PACT-cleared tissue) showing distribution of EGFP-labeled (green) and tdTomato-labeled (magenta) neurons. Scale bar, 200 μm.

As with the mPFC injections, CAV2-cre injection in the M1 of TrAC-LC mice labeled a greater percentage of LC-NE neurons on the ipsilateral side. We counted 108 ± 28 M1-projecting (EGFP+) NE neurons and 571 ± 42 tdTomato+ NE neurons ipsilateral, and 12 ± 3 M1-projecting (EGFP+) NE neurons and 617 ± 55 tdTomato+ NE neurons contralateral to the injection site. Thus, CAV2-Cre injection in M1 labeled 15.19 ± 3.16% of the ipsilateral LC and 1.85 ± 0.39% of the contralateral. Of the retrogradely labeled neurons in LC, 90% (108/120) were ipsilateral to the M1 injection site and 10% (12/120) were contralateral to the injection site. Again, this distribution is similar to that reported previously for LC projections to cortical targets using conventional retrograde tracer techniques (Simpson et al., 1997; Chandler and Waterhouse, 2012; Chandler et al., 2014). As observed following injection in mPFC, the EGFP-labeled neurons were distributed throughout the rostrocaudal extent of the anatomically defined LC (Fig. 4). Unlike the mPFC-projecting neurons, however, the percentage of M1-projecting (EGFP+) NE neurons was similar in the LC core and rostral extension (20.03 ± 4.12% rostral extension, 19.49 ± 5.82% core; *p* = 0.9418^b^, unpaired *t* test; Table 1). M1-projecting neurons, like mPFC-projecting neurons, were generally absent from subcoeruleus/A7 (one EGFP+ neuron in one mouse).

**Figure 5.**
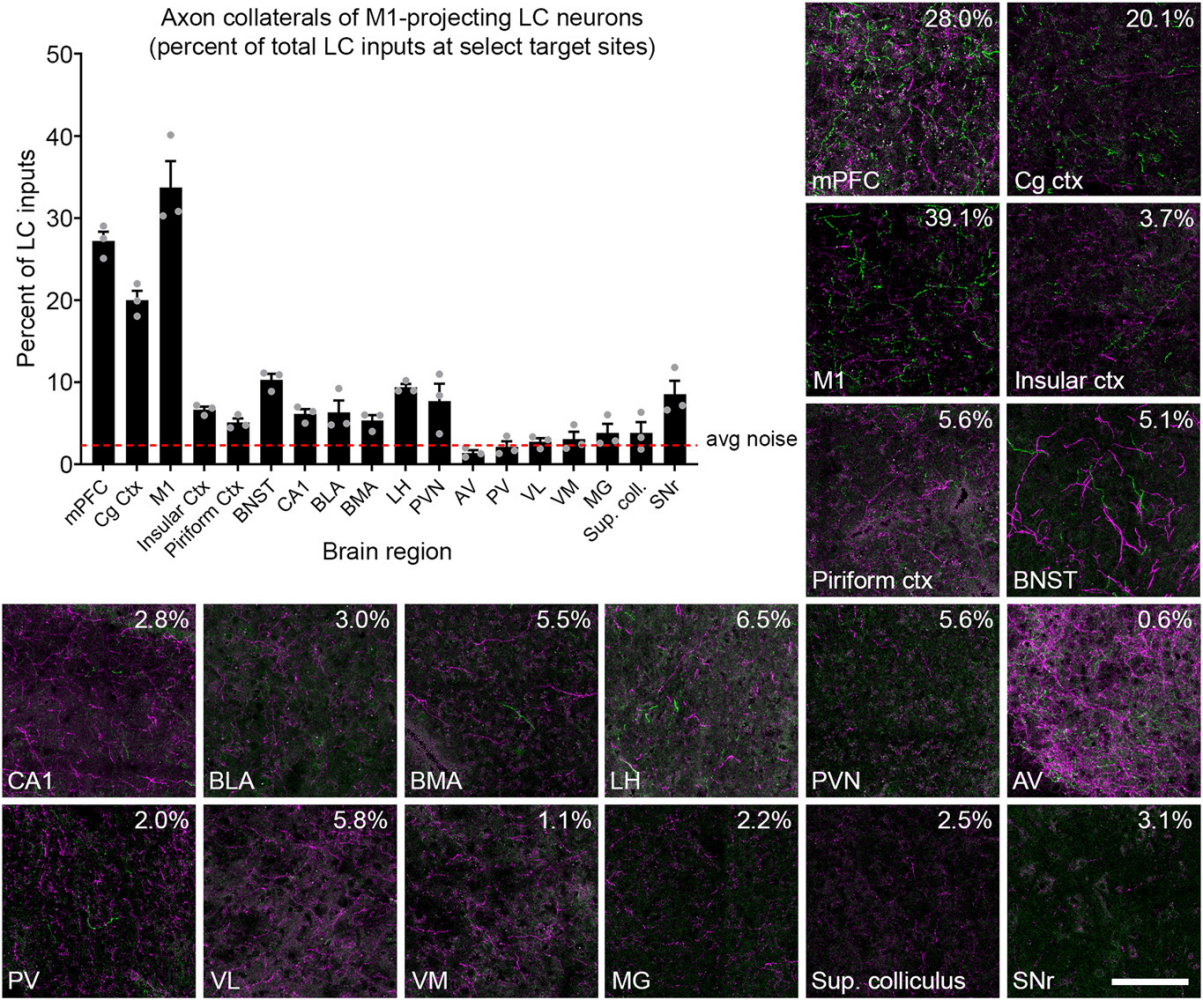
Distribution of axon collaterals from LC neurons projecting to motor cortex. Representative fluorescent images show Cre+ (EGFP-labeled, green) and Cre-negative (tdTomato-labeled, magenta) axons in select forebrain and midbrain regions of a TrAC-LC mouse injected with CAV2-Cre in M1. Numbers in the upper right corner of each image indicate the percent of LC-NE axons in the image that are EGFP+. The bar graph (*n* =* *3 mice) indicates the percentage of LC-NE axons at each target region that originate from EGFP+ M1-projecting LC-NE neurons (are mean ± SEM). The dotted line represents the noise in the green channel (average green pixel count in areas qualitatively lacking EGFP-labeled axons, divided by average total pixel count). Percentages below the dotted line are indistinguishable from noise. The scale bar for fluorescent images indicates 100 μm, and each image represents 13% of the volume quantified in the bar graph.

When we examined the distribution of axon collaterals from EGFP+ M1-projecting LC-NE neurons as a percentage of all LC inputs at target sites (Fig. 5), we again observed the highest percentage of labeled axons near the injection site (M1; 33.7 ± 3.2%). As with the mPFC injections, we observed widespread EGFP+ axon collaterals in cortical and subcortical regions, with percent contributions ranging from 27.2 ± 1.1% in mPFC to 1.4 ± 0.3% in anteroventral thalamic nucleus (AV). Outside the cortex, the highest percent contribution of EGFP+ axons was observed in the BNST (10.3 ± 0.7%) and lateral hypothalamus (LH; 9.4 ± 0.4%). As with the mPFC-projecting subpopulation, axon collaterals from M1-projecting neurons constituted a small percentage of all LC-NE inputs to thalamic nuclei (Fig. 5). Although contribution from mPFC-projecting neurons was consistently above the average noise (Fig. 3), that from the M1-projecting neurons was at or below the average noise level in all thalamic nuclei that we examined (Fig. 5). This very sparse innervation of the thalamus by the M1-projecting LC subpopulation is notable, given that the LC is the major source of noradrenergic inputs to many thalamic nuclei (Robertson et al., 2013), including several that we examined (e.g., AV, VL, VM). In thalamic regions that receive significant non-LC noradrenergic input (e.g., PV; Robertson et al., 2013), collaterals from the M1-projecting LC subpopulation would represent a particularly small fraction of noradrenergic inputs.

**Figure 6.**
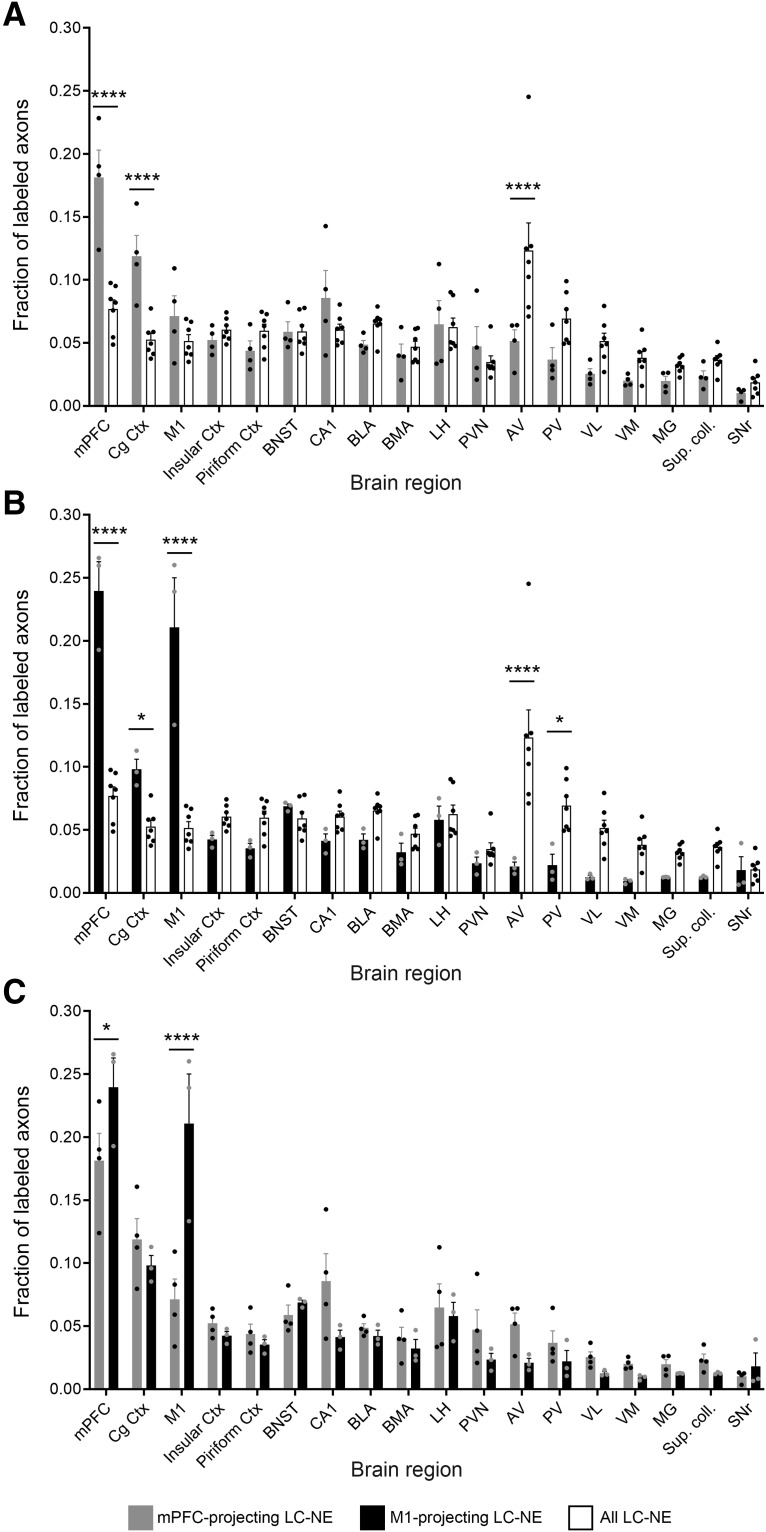
Distribution of axon collaterals from mPFC-projecting and M1-projecting LC-NE neurons differs from that of the LC as a whole and from each other. Bar graphs show fractional distribution of labeled mPFC-projecting, M1-projecting, or all LC-NE neurons at select target regions. Axon density at each region is represented as a fraction of labeled axons in all imaged regions. Bar graph data are mean ± SEM and were analyzed by two-way repeated measures ANOVA. ***A***, Comparison of mPFC-projecting (*n* = 4 mice, EGFP+) and all LC-NE neurons (*n* = 7 mice, sum of EGFP+ and tdTomato+ axons). LC subpopulation by target region interaction: *F*_(17,153)_ = 8.072, *p* < 0.0001. Bonferroni *post hoc* test, ****p < 0.0001 [95% confidence interval of difference (CI of diff.): mPFC, −0.1441 to −0.06482; Cg Ctx, −0.1059 to −0.02655; AV, 0.03218 to 0.1115]. ***B***, Comparison of M1-projecting (*n* = 3 mice, EGFP+) and all LC-NE neurons (as above). LC subpopulation by target region interaction: *F*_(17,136)_ = 21.87, *p* < 0.0001. Bonferroni *post hoc* test, *p = 0.0176, Cg Ctx (95% CI of diff.: −0.08,684 to −0.004373), *p = 0.0117 PV (95% CI of diff.: 0.005982 to 0.08845), ****p < 0.0001 (95% CI of diff.: mPFC, −0.2037 to −0.1213; M1, −0.2007 to −0.1182; AV, 0.06125 to 0.1437). ***C***, Comparison of mPFC-projecting and M1-projecting LC-NE neurons. Injection site by target region interaction: *F*_(17,85)_ = 5.152, *p* < 0.0001. Bonferroni *post hoc* test, *p < 0.0217 (95% CI of diff.: 0.004639 to 0.1114), ****p < 0.0001 (95% CI of diff.: 0.08626 to 0.1930).

As an alternative method for analyzing the collateral data, we calculated the fractional distribution of axon collaterals across target regions (i.e., the fraction of a population of axons that innervate each target site). This method has previously been used in retrograde viral analysis of LC projections where the uninfected LC subpopulation was not labeled (Schwarz et al., 2015; Uematsu et al., 2017). Because our analysis, similar to the prior studies, quantifies a fixed volume in brain regions that differ dramatically in size and shape, this analysis reveals relative axon densities, not total axon numbers, in the brain regions. In the present study, with the whole LC labeled, we were able to compare the fractional distribution of projections from each retrogradely labeled subpopulation with that of the LC as a whole (Fig. 6*A*,*B*). Compared with the retrogradely labeled subpopulations, we observed the LC as a whole projecting more uniformly to cortical and subcortical regions, although with a notably large fraction in the anteroventral thalamic nucleus. When we directly compared the distribution of axon collaterals labeled by mPFC or M1 injection (Fig. 6*C*), we observed the fraction of EGFP+ axons to be significantly different at the injection sites (*p* = 0.0217^c^ mPFC and *p* < 0.0001^d^ M1; Table 1). Following injection in M1, a surprisingly large fraction of EGFP+ axons were observed in mPFC (Fig. 6*C*), a region which may include both collaterals of M1-projecting neurons and axons of passage projecting to more caudal cortical regions (Morrison et al., 1978, 1979). A greater fraction of efferents from mPFC-projecting LC-NE neurons appear to innervate the thalamus; although the difference did not reach statistical significance in individual regions that were imaged, the sum of fractional innervation of the five thalamic subregions was significantly greater for the mPFC-projecting neurons (0.1534 ± 0.0211 mPFC, 0.0773 ± 0.0119 M1; *p* = 0.0367^e^, unpaired *t* test; Table 1). Taken together, these results suggest that not only are different brain regions (e.g., M1 and mPFC) innervated by different subpopulations of LC-NE neurons, but those subpopulations differ from each other and the larger LC-NE system in their pattern of axon collateralization.

## Discussion

The noradrenergic LC represents an extreme example of the ability of projection neurons to form collateral branches. From a small population of neurons, ∼2300 in the bilateral mouse LC (Berger et al., 1979; Touret et al., 1982; Plummer et al., 2017), a dense network of axonal fibers extends throughout the brain and spinal cord. While it is clear that individual LC-NE neurons send collaterals to multiple terminal fields, conventional tracing methods are not capable of identifying the degree or pattern of collateralization in great detail. For example, injecting multiple retrograde tracers into discrete terminal fields to identify all structures co-innervated by specific subsets of LC cells is impractical since tracer uptake can be unpredictable, as well as too labor intensive for large scale analyses. Recently, two newer strategies have been applied to the LC-NE system. The first, MAPSeq, is a high-throughput sequencing-based approach that provides information about axonal collateralization at single cell resolution, but lacks any information on the spatial organization of LC neurons projecting to a given target or their axonal architecture (Kebschull et al., 2016). The second strategy retains spatial information by employing a dual viral approach to fluorescently label LC neurons projecting to a target brain region. This approach requires retrograde delivery of a recombinase from the target structure and a recombinase-dependent reporter delivered to the LC (Schwarz et al., 2015; Uematsu et al., 2017). The addition of a second viral injection increases the chance of variable targeting, particularly when there is no foolproof method to confirm that the virally delivered reporter is expressed in all LC-NE neurons. While this method can be used successfully to target compact, anatomically discrete structures like the LC, it is of limited value for the study of dispersed cell populations.

TrAC, on the other hand, defines the cell population of interest genetically (without virus) and is not restricted by the distribution of the retrogradely labeled population, making it amenable to the study of dispersed populations. Moreover, the cell population of interest is not limited to cells that are defined by adult gene expression, as is the case with viral labeling. Instead, the population of interest can be defined using transient developmental genetic information. As shown in this article, if the fluorescent indicator allele used in TrAC is capable of simultaneously labeling Cre+ and Cre-negative populations, the retrogradely labeled neurons can be directly observed in the context of the genetically defined population. In the case of complex, heterogeneous neuronal populations such as the central noradrenergic system, use of the triple recombinase responsive allele *RC::RFLTG* (Plummer et al., 2015) allows analysis to be restricted to a subset of a broader neuronal population. Thus, TrAC allowed us to observe LC axons in isolation at brain targets (e.g., BNST, PVN) that also receive significant inputs from other noradrenergic nuclei. Importantly, this method has allowed us to reliably recapitulate anatomic features that have been previously demonstrated using conventional tracers (e.g., a primarily ipsilateral projection from LC to cortex; Foote et al., 1980; Loughlin et al., 1986; Simpson et al., 1997; Chandler et al., 2014; Figs. 2, 5), while simultaneously revealing previously inaccessible features of axonal collateralization that could not be revealed by tracer molecules.

The data presented in the current study argue for neither restricted and discrete efferent connectivity between LC neurons and their terminal fields (Chandler et al., 2013, 2014), nor for a global broadcast model in which all LC neurons innervate most or all terminal fields (Aston-Jones et al., 1986; Schwarz et al., 2015). Instead, they are broadly consistent with previous analyses (Kebschull et al., 2016; Uematsu et al., 2017) indicating that axons of LC projection neurons tend to have a major terminal field destination which they densely innervate, and widespread minor terminal field targets which contain sparser axonal collaterals than the primary preferred region. Consistent with this model, we found that LC neurons projecting to mPFC have a moderate to sparse network of collaterals to other parts of the cortex and various subcortical locations. While the same generally holds true for cells that project to M1 cortex, one apparent deviation from this trend comes from the observation that an injection of CAV2-CMV-Cre into M1 resulted in a significantly greater fraction of EGFP+ axons in mPFC than an injection of the virus into mPFC (Fig. 6*B*). While this result seems paradoxical, multiple explanations are possible. One possibility is that the labeled axons observed in mPFC after injection in M1 include fibers of passage en route to M1. Another potential explanation, which does not preclude the first, is that LC neurons innervating M1 collateralize to fewer brain regions than do those innervating mPFC, resulting in a larger fraction of the total collaterals from M1-projecting neurons localized in a few target regions, with smaller fractions elsewhere. This possibility is supported by the observation that injections into M1 resulted in a smaller fraction of EGFP+ axons in the thalamus as a whole, relative to injection in mPFC.

A question which arises from this analysis is whether or not the sparser axons that occur outside of the “primary” target do so in some organized fashion with a functional consequence, i.e., could the coordinated release of NE in mPFC and other brain regions by way of axonal collateralization have some operational role in brain function and behavior? It has been previously reported that individual LC neurons collateralize to innervate multiple structures along an ascending sensory pathway (Simpson et al., 1997), possibly as a means of simultaneously promoting NE release throughout a functional network to facilitate sensory encoding. TrAC therefore represents a viable method of identifying networks among which release of NE or other LC co-transmitters might occur simultaneously to facilitate a specific neurophysiological or behavioral functional role. The functional organization of LC-NE axon collaterals to cognitive, sensory and motor terminal fields can now be probed using this approach.

The difference in the rostrocaudal distribution of mPFC-projecting and M1-projecting neurons suggests that they constitute distinct, although overlapping, subpopulations of LC-NE neurons. However, our current inability to simultaneously label neurons projecting to two different injection sites precludes determination of the precise degree of overlap between the two subpopulations. Such analysis may become possible with a new generation of tools, including CAV2 vectors expressing recombinases other than Cre and indicator alleles like *RC::RFLTG* that express different fluorescent proteins in response to combinatorial recombinase expression. Nevertheless, the current TrAC strategy, by simultaneously labeling a genetically defined neuronal population and a projection-based subpopulation, permits analyses of axon collateralization that have not previously been practicable in complex genetically and neurochemically defined neuronal subtypes.

## Synthesis

Reviewing Editor: Lynda Erskine, University of Aberdeen Decisions are customarily a result of the Reviewing Editor and the peer reviewers coming together and discussing their recommendations until a consensus is reached. When revisions are invited, a fact-based synthesis statement explaining their decision and outlining what is needed to prepare a revision will be listed below. The following reviewer(s) agreed to reveal their identity: Naohiro Koshiya, Carolyn Harley.

As you will see, both referees express interest in your work, which provides both a worthwhile methodological advance and novel insights into specific aspects of locus coeruleus connectivity. However, it was agreed that the manuscript would benefit from additional detail on some of the approaches used as well as additional analyses of some of the data sets. Appended below are the points from the reviewers that, following consultation between myself and the reviewers, we have agreed are essential to address:

-----------------------------

This paper, “An intersectional viral-genetic method for fluorescent tracing of axon collaterals reveals details of noradrenergic locus coeruleus structure,” demonstrates the capability of their new TrAC (Tracing Axon Collaterals) genetic neuroanatomical strategy. The noradrenergic/ norepinephrinergic (NE) neurons of Locus Coeruleus (LC) have been known to project throughout the brain. The question here was whether each LC-NE neuron projects to wide regions unselectively. The answer according to this study is no in some case. Some of LC-NE neurons projected differentially to the medial prefrontal cortex (mPFC) and primary motor cortex (M1) as they don’t fully share collateralized projections from LC.

In classical method, to determine shared or differential projections from one source population, double retrograde fluorophore labeling from target regions, e.g., mPFC and M1, to cell bodies of the population of interest, e.g., LC, would be used. The present TrAC is new to show collaterals themselves from one target region to the other via the common upstream axons and cell bodies. The beauty here is the mother population LC phenotype was simultaneously defined with En1 and Dbh transient expression mechanisms so that terminal fibers are labeled with different fluorescent proteins: EGFP for collaterals shared with the injected target region or tdTomato for negative of that. Thus the TrAC is well specialized for axon collateral-based common/ differential projection receptions (as indeed the acronym is defined).

The novel technique described permits more accurate characterizations of the brain connectome for all neuronal groups for which suitable transgenic animals are available. The manuscript itself clarifies questions that have recently been raised about the specificity of LC projections and about the likely existence of discrete functional ensembles in LC. It is consistent with earlier work, but provides novel insights into specific aspects of LC connectivity as well as raising its own questions.

Major

(A): In Abstract, Significant Statement, and elsewhere in the text, visualization of projection-based subpopulation of LC-NE neuron cell bodies is listed as a major feature. Their examples are crisply shown as green cells among magenta population in Figures 2 and 4 for mPFC- and M1-projecting LC-NE neurons, respectively. The paper title also states, “details of noradrenergic locus coeruleus structure.” These could be better addressed in the text. The two subpopulations are only tersely documented in Results for their distributions within the LC-NE population, and the only difference between the two might be inclusion (mPFC-projecting) or not (M1-projecting) of the subcoeruleus. No quantitative spatial differences within LC proper? Don’t they have different topographic distributions against the entire LC-NE population? Since the two subpopulations are visualized in excusive fashion in different preparations, comparison of their distributions could be only possible against common mother population. Such reference populations are indeed available probably for the first time with the present “all” LC-NE (magenta/ tdTomato) labeling, so, they could be utilized to address the comparison. If not, the major feature as well as the title phrase might lack substance, would be misleading, and might need to be down stated, deleted, or replaced. Since the study utilized the thick, clarified tissue visualization with PACT method, or with digitized stack of serial sections, some sort of quantitative spatial comparison of the two sets of mPFC- and M1-projecting neurons over normalized LC-NE population could be made. Three-axis distribution histogram comparison (rostrocaudal, dorsoventral, and mediolateral) through the LC-NE extent with a three-dimensional rendering (cf. Plummer et al, 2015, Figure 5) for example might substantiate the major feature of TrAC. These could be addressed if digitized 3-D data are kept and can be quantified semi-automatically by Fiji.

(B): Axon collateral distributions were quantified for fixed-sized tissue volume, by pixel count in maximum intensity projection image. The present comparisons would only be valid when choices of volumes are relevant each other; the results are totally dependent on the choices. Objective criteria on how the particular regions’ volumes were selected could have been set and could be stated.

Minor

(C): Methods: It would be useful if the position of the head in the stereotaxic were indicated. Was it skull flat? Height difference could be stated between either Lambda and Bregma or the inter-oral line and the bite-bar.

(D): Qualitative differences of 40-um frozen sections and 3-mm thick PACT cleared tissue, both of which were reportedly used but unclearly, could be documented (e.g., difference in image quality, or which is used for which Figure pictures) as well the quantities of them (how many each was used for those bar graphs).

(E): Related to Major (B), terminal fiber quantification (graph) and image data may currently not be consistent. For example, Figure 3’s Cg Ctx image may have more Green over Magenta while according to the accompanied graph, it is at most less than 40%. How much is the ratio in the particular image? Perhaps each image can have the number besides region name or in the legend. The array of images is otherwise not informative. The same thing may apply to Figure 5.

(F): On Line 361 you might consider omitting ‘much’ before sparser.

(G): In Figure 6, horizontal bars that indicate pairs of significant difference with stars could be repositioned leftward to over the pairs of panel A, AV, and panel C, M1.
